# Acute and chronic phases of complex regional pain syndrome in mice are accompanied by distinct transcriptional changes in the spinal cord

**DOI:** 10.1186/1744-8069-9-40

**Published:** 2013-08-08

**Authors:** Joseph J Gallagher, Maral Tajerian, Tianzhi Guo, Xiaoyou Shi, Wenwu Li, Ming Zheng, Gary Peltz, Wade S Kingery, J David Clark

**Affiliations:** 1 Anesthesiology Service, Veterans Affairs Palo Alto Health Care System, 3801 Miranda Ave., Palo Alto, CA, 94304, USA; 2 Department of Anesthesiology, Stanford University School of Medicine, Stanford, CA, USA; 3 Physical Medicine and Rehabilitation Service, Veterans Affairs Palo Alto Health Care System, Palo Alto, CA, USA

**Keywords:** Complex regional pain syndrome, CcL2, Chemokine, Chronic pain, Spinal cord, Microarray analysis, Transcriptome, Pathway analysis

## Abstract

**Background:**

CRPS is a painful, debilitating, and often-chronic condition characterized by various sensory, motor, and vascular disturbances. Despite many years of study, current treatments are limited by our understanding of the underlying mechanisms. Little is known on the molecular level concerning changes in gene expression supporting the nociceptive sensitization commonly observed in CRPS limbs, or how those changes might evolve over time.

**Results:**

We used a well-characterized mouse tibial fracture/cast immobilization model of CRPS to study molecular, vascular and nociceptive changes. We observed that the acute (3 weeks after fracture) and chronic (7 weeks after fracture) phases of CRPS-like changes in our model were accompanied by unique alterations in spinal gene expression corresponding to distinct canonical pathways. For the acute phase, top regulated pathways were: chemokine signaling, glycogen degradation, and cAMP-mediated signaling; while for the chronic phase, the associated pathways were: coagulation system, granzyme A signaling, and aryl hydrocarbon receptor signaling. We then focused on the role of CcL2, a chemokine that we showed to be upregulated at the mRNA and protein levels in spinal cord tissue in our model. We confirmed its association with the nociceptive sensitization displayed in this model by demonstrating that the spinal but not peripheral administration of a CCR2 antagonist (RS504393) in CRPS animals could decrease mechanical allodynia. The spinal administration of CcL2 itself resulted in mechanical allodynia in control mice.

**Conclusions:**

Our data provide a global look at the transcriptional changes in the spinal cord that accompany the acute and chronic phases of CRPS as modeled in mice. Furthermore, it follows up on one of the top-regulated genes coding for CcL2 and validates its role in regulating nociception in the fracture/cast model of CRPS.

## Background

Complex regional pain syndrome (CRPS) is a painful, debilitating, and often-chronic condition with an estimated incidence rate of 26.2 per 100,000 person years [1]. While acute CRPS sometimes improves with early and aggressive physical therapy, CRPS present for a period of one year or more seldom spontaneously resolves. The syndrome encompasses a disparate collection of signs and symptoms involving the sensory, motor and autonomic nervous systems, cognitive deficits, bone demineralization, skin growth changes and vascular dysfunction [2]. Current therapies for CRPS including physical, interventional, pharmacological, rehabilitative and alternative are limited in their effectiveness, and none are routinely curative of the chronic condition [3,4]. The acute phase of the condition is often characterized by edema and warmth, and is thought to be supported by neurogenic inflammation [5-7]. Alterations in CNS structure and function may be more important to the sustained pain and neurocognitive features of the chronic phase of the CRPS [8].

The molecular analysis of peripheral mechanisms supporting CRPS has been extensive. Human studies have focused on dysfunctional signaling through autonomic and peptidergic neurons [9-11]. Other work has shown abnormal levels of cytokines in the skin of CRPS limbs [12]. More recently changes in the adaptive system of immunity have been demonstrated [13]. To better understand these changes, a tibial fracture/cast immobilization model of CRPS has been developed in rodents displaying nociceptive sensitization, bone demineralization, edema and warmth [14,15]. This model also recapitulates the human observations of abnormal peripheral neural signaling and cytokine generation, particularly in the first several days after removal of cast immobilization. The fracture/cast model has not to this point been utilized for the purpose of understanding the more chronic features of CRPS, though nociceptive changes are persistent for months in these animals.

The development of pain involves a complicated sequence of events ranging from changes in neuronal properties [16] to alterations in gene transcription and protein levels [17,18]. While many successes have been achieved through the selection of individual molecules for study as they participate in pain, a complementary approach has been to study changes in the expression levels of large numbers of genes in hypothesis-free fashion using expression arrays and similar molecular tools. A recent meta-analysis of pain related gene expression studies on spinal cord and dorsal root ganglion tissue revealed both similarities and differences across a range of pain models [19]. These authors discovered that two genes, Reg3b (regenerating islet-derived 3 beta; pancreatitis-associated protein) and CcL2 (chemokine [C-C motif] ligand 2), were up-regulated in almost every dataset, included in their analysis, suggesting possible core roles in supporting persistent pain.

To this point no array-based studies have been provided using a model of CRPS, a condition which has features separate from most acute, inflammatory and neuropathic etiologies of pain [10]. Furthermore, the striking transition of CRPS from an acute to a more chronic state suggests that analyses need to be conducted at more than one timepoint. Very few array-based analyses have commented on changes in gene expression over time. We hypothesized that array-based spinal cord gene expression studies using the fracture-cast model of CRPS would reveal timepoint-dependent genes and pathways relatively unique to the CRPS model, that a set of core genes might be shared with other models of persistent pain, and that at least one significantly changed gene could be shown to be functionally related to nociceptive sensitization in this model.

## Results

### CRPS mice exhibit transient increases in temperature and edema in addition to long-lasting nociceptive sensitization in the injured limb

**Figure 1:**
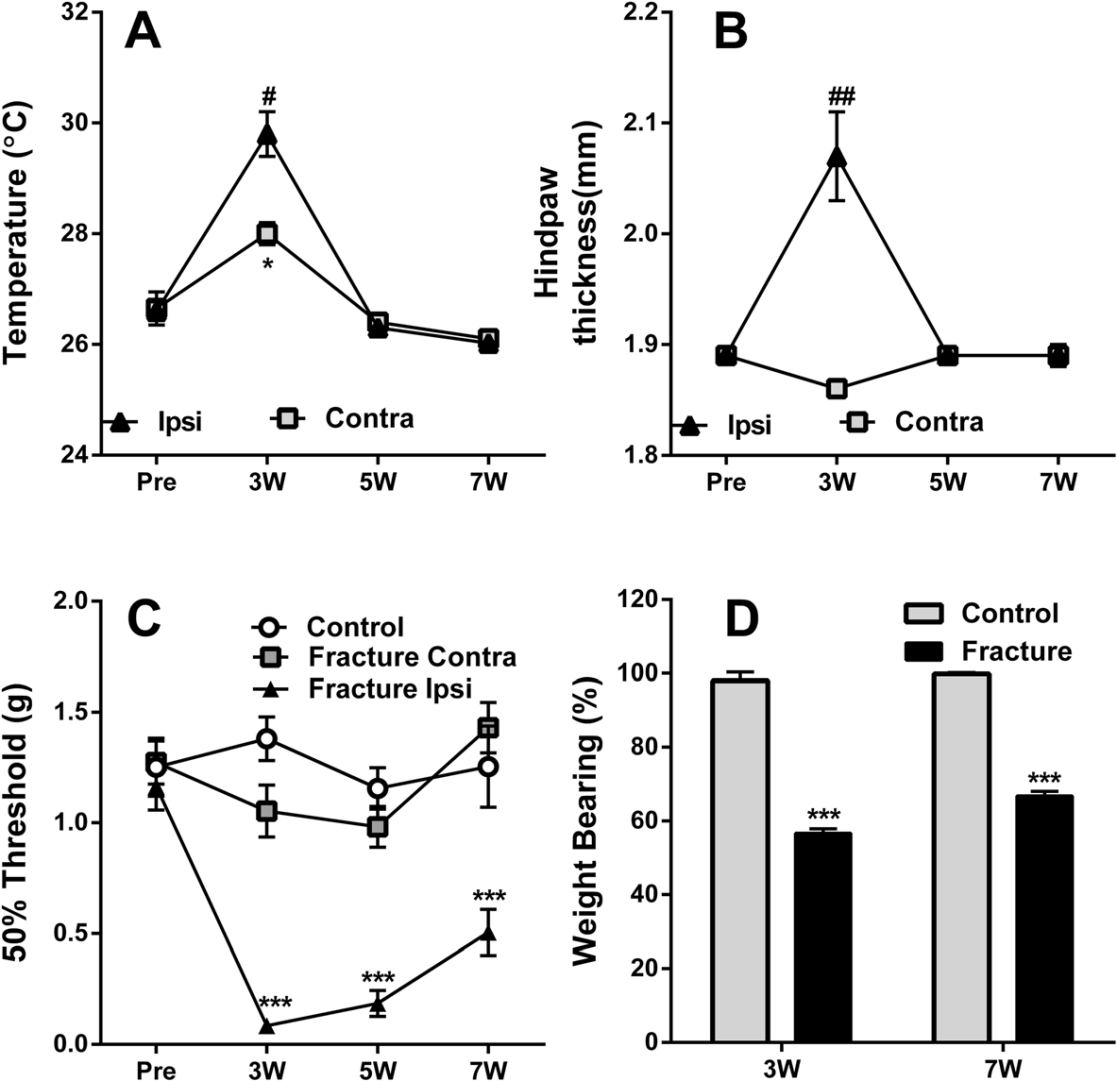
**Physiological and behavioral changes in CRPS mice.** CRPS mice display increased temperature **(A)** and edema **(B)** on the affected hindpaw at 3 weeks post-fracture. In addition, they show signs of mechanical allodynia **(C)** and decreased weight bearing **(D)** for up to 7 weeks after fracture. **p<0.01, *** p<0.001. n=8/group. Errors bars=S.E.M.

Ipsilateral and contralateral measurements of hindpaw temperature and edema, two commonly observed symptoms in CRPS patients, were performed at 3, 5 and 7 weeks post-fracture. Both temperature and edema demonstrated similar profiles, exhibiting transients increases identified at the 3-week, but not the 5- or 7-week timepoints. An increased temperature in the contralateral hindpaw, as compared to baseline, was observed at the 3-week timepoint only (Figure 1A,B). Mechanical hypersensitivity, assessed using Von Frey filaments, identified a significant and persistent reduction – up to 7 weeks post-fracture - in the injured hindpaw compared to measurements of the contralateral hindpaw or control mice. Inspection of the mechanical hypersensitivity of the contralateral hindpaw suggested a modest decrease that did not reach significance at the 3 and 5 weeks (Figure 1C). Reduced weight bearing was present in the injured hindpaw and persisted at both timepoints assessed, extending from 3 to 7 weeks after fracture (Figure 1D).

### Microarray expression analysis from ipsilateral spinal cord identifies distinct profiles at 3 and 7 weeks post-fracture

We identified the genes significantly regulated at the 3- and 7-week timepoint, with respect to the control group (absolute fold change > 1.5, adjusted p-value < 0.05). 199 genes were identified as significantly regulated at the 3-week timepoint with 98 of these genes also identified as significantly regulated at the 7-week timepoint. 62 genes were unique to the 7-week timepoint. Half of the 122 genes with increased expression at the 3 week timepoint maintained increased expression at the 7-week timepoint (61 genes), while 38 of the 97 genes with decreased expression at the 3-week timepoint maintained decreased expression at the 7-week timepoint with the remaining genes showing no differential expression at the 7-week timepoint. No genes were identified as reversing their expression from over expressed to under expressed or vice versa between timepoints.154 genes are significantly regulated at the 7-week timepoint with 103 genes increased and 51 genes decreased (Additional file 1: Table S1; Additional file 2: Table S2).

### CRPS results in altered transcriptional programs in the spinal cord

**Figure 2:**
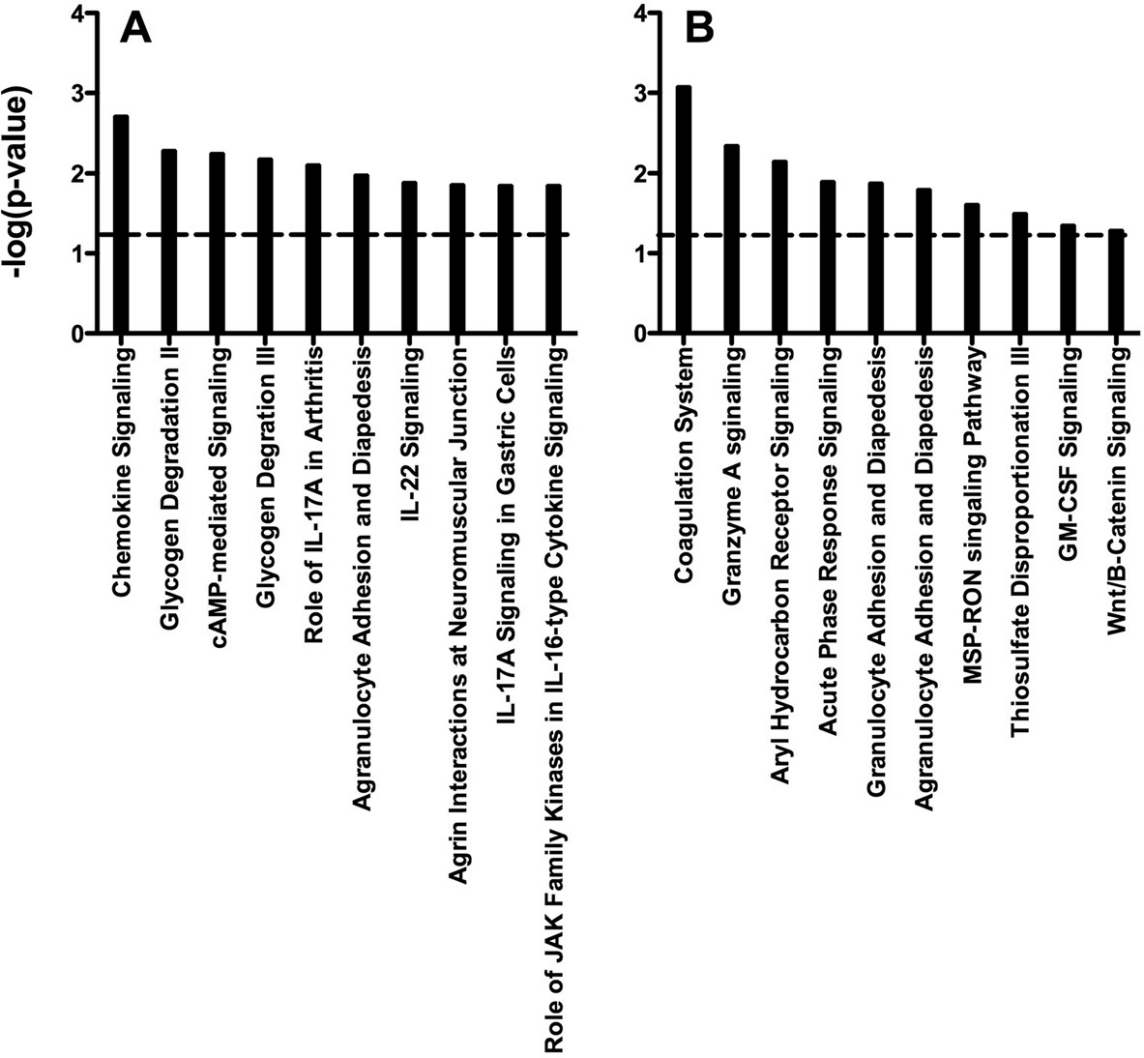
**Canonical pathway analysis.** CRPS affects transcriptional programs unique to each of the acute and chronic timepoints. All pathways were scored and ranked according to Ingenuity Pathway Analysis using Fisher’s exact test. The dotted line indicates the threshold value of p<0.05.

Ingenuity Pathway Analysis (IPA) identified specific networks that were dysregulated in the spinal cord 3 and 7 weeks following fracture. Canonical pathways with a cutoff p-value < 0.05 were considered statistically significant. Canonical pathways, indicating wide changes in currently known pathways, were shown to differ between the two timepoints. At 3 weeks, the main regulated pathways were chemokine signaling, glycogen degradation II, cAMP-mediated signaling, glycogen degradation III, role of IL-17A in arthritis, agranulocyte adhesion and diapedesis, IL-22 signaling, agrin interactions at neuromuscular junction, IL-17A signaling in gastric cells, and role of JAK family kinases in IL-6-type cytokine signaling. At the 7-week timepoint, the effected pathways were: coagulation system, granzyme A Signaling, aryl hydrocarbon receptor signaling, acute phase response signaling, granulocyte adhesion and diapedesis, agranulocyte adhesion and diapedesis, MSP-RON signaling pathway, thiosulfate disproportionation III (Rhodanese), GM-CSF signaling, and agrin interactions at neuromuscular junction (Figure 2). In addition to these canonical pathways, we identified the following top biological functions associated, to different degrees, with both timepoints: Cellular movement, cancer, cardiovascular system development and function, organismal development, nutritional disease, cell death and survival, nucleic acid metabolism, small molecule biochemistry, cell-to-cell signaling and interaction, molecular transport, hematological system development and function, immune cell trafficking, inflammatory response, tissue development, cellular function and maintenance, cell-mediated immune response, cellular development, lipid metabolism, cell morphology, hypersensitivity response (Additional file 3: Table S3).

**Figure 3:**
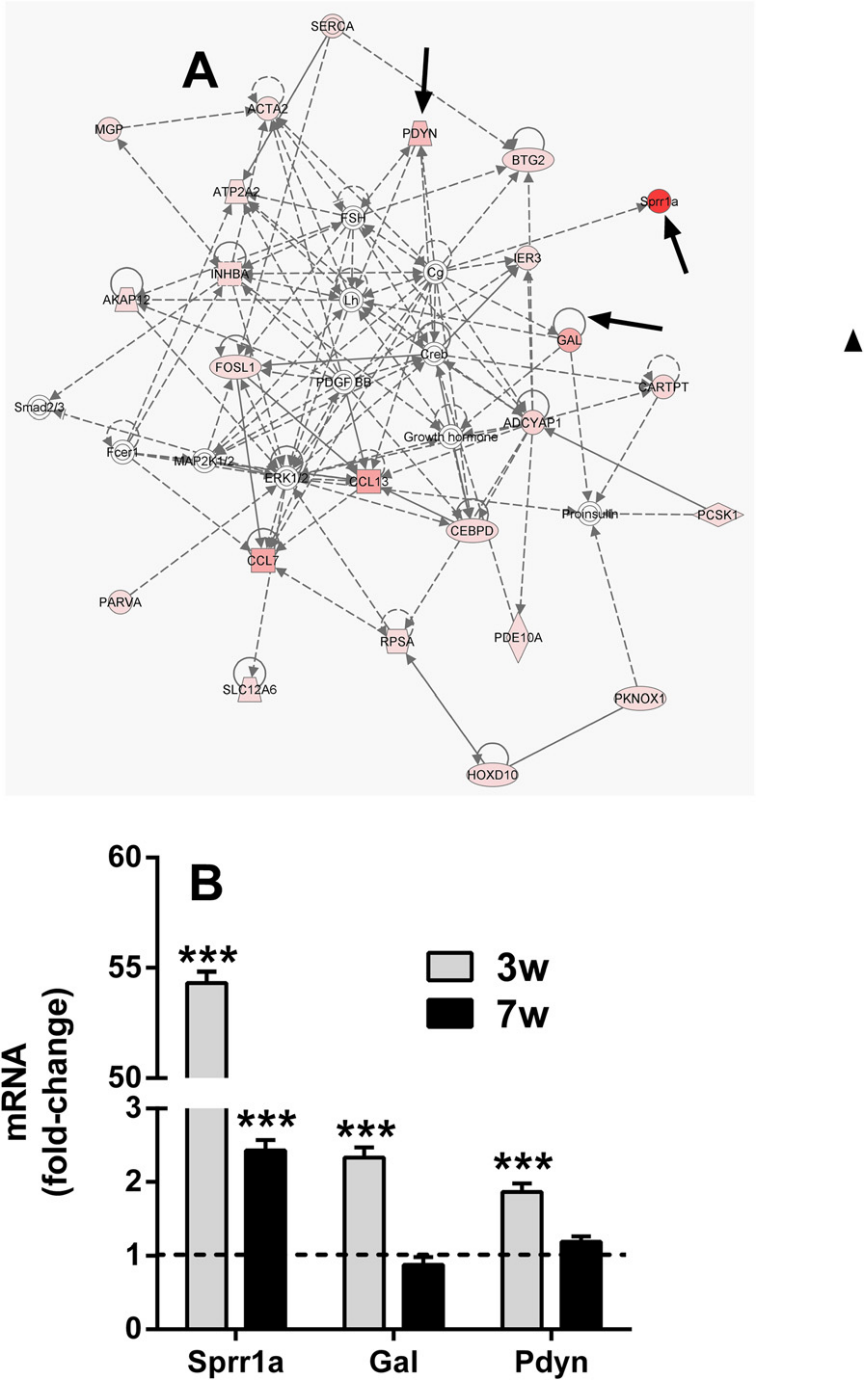
**Transcriptional pathway analysis and validation of transcript mRNA expression.** IPA identified interacting networks affecting cell signaling. Up-regulated transcripts are marked with red **(A)**. SPRR1a, GAL, and PDYN transcripts were validated by quantitative PCR. The dotted line indicates control measures **(B)**. *** p<0.001. n=4/group. Errors bars=S.E.M.

Furthermore, qPCR measures of transcript expression allowed us to validate the upregulation of some of the genes (SPRRA1a, GAL, and PDYN) involved in a cell-to-cell signaling pathway (Figure 3A,B), a pathway with particular relevance to nociceptive signaling. Additional validation transcripts are shown in Additional file 4: Figure S1A,B. No changes were observed in the expression of astrocyte and macrophage markers, GFAP and Iba1, respectively (Additional file 4: Figure S1C).

### Spinal CcL2 mRNA and protein levels are increased at 3 but not 7 weeks post-fracture

**Figure 4:**
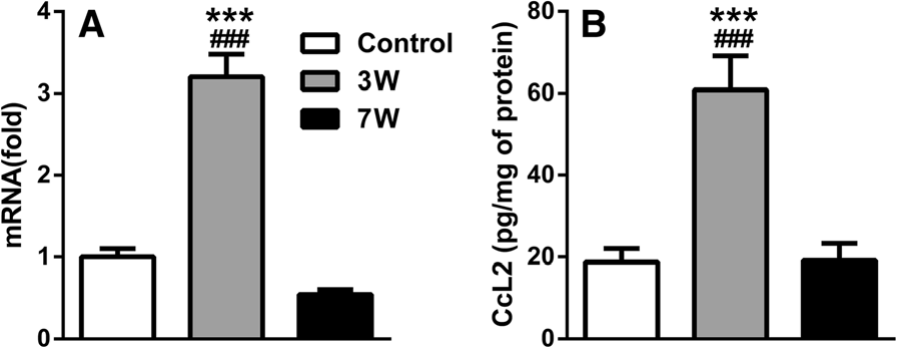
**Increase in spinal CcL2 mRNA and protein levels in CRPS mice.** 3 weeks following fracture, CRPS mice show a significant increase in spinal CcL2 levels at the mRNA **(A)** and protein **(B)** levels, compared to both control (*) and the 7-week timepoint (#). *** p<0.001. n=4/group. Errors bars=S.E.M.

Both our array data and previous array studies have identified CcL2 as a possible common chronic pain gene. CcL2 mRNA levels were increased at the 3- but not 7-week timepoint (Figure 4A). Similarly, CcL2 protein levels were increased at the 3-week timepoint only (Figure 4B).

### Intrathecal, but not intraplantar, administration of a CCR2 antagonist leads to transient reduction in mechanical hypersensitivity 3 and 7 weeks post-fracture

To assess the role of CcL2 signaling in pain behavior, we pharmacologically blocked a major CcL2 receptor (CCR2) at 3 and 7 weeks post-fracture using a CCR2 antagonist, RS504393.

**Figure 5:**
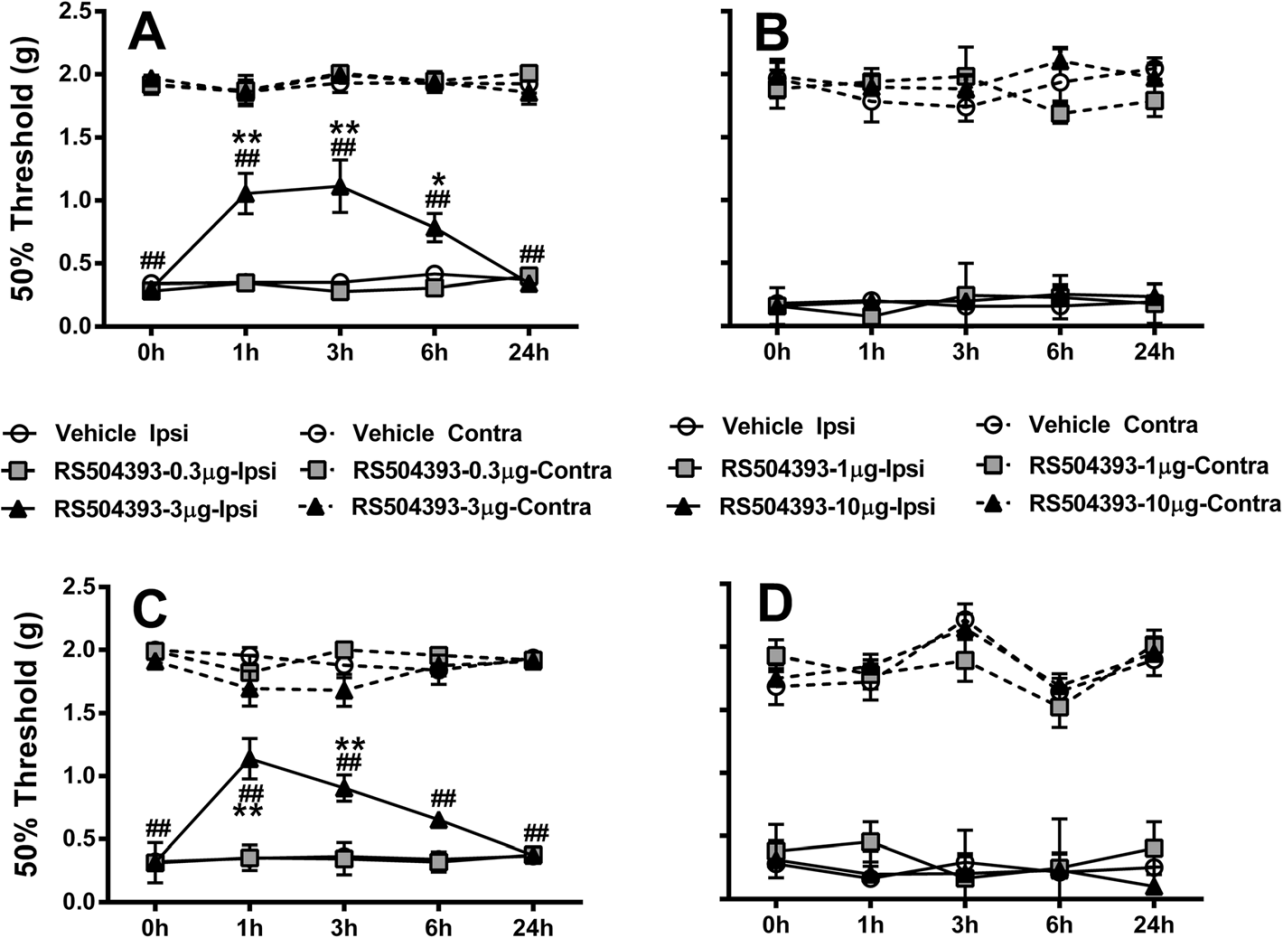
**Amelioration of mechanical allodynia in CRPS mice following intrathecal, but not intraplantar, administration of the CCR2 antagonist, RS504393.** Intrathecal RS504393 (3 μg) resulted in increased mechanical thresholds in the affected paw compared to vehicle-treated (*) and control mice (#). This was true for both the 3- **(A)** and 7-week **(C)** timepoints. Intraplantar injections failed to show any efficacy **(B,D)**. * p<0.05; ** p<0.01.n=8/group. Errors bars=S.E.M.

Intrathecal administration of the CCR2 antagonist at the high dose (3 μg) led to statistically significant reductions in mechanical hypersensitivity 1, 3 and 6 hours after injection 3 weeks post-fracture; statistically significant reductions 7 weeks post-fracture were observed 1 and 3 hours after injection but did not reach significance at the 6 hour timepoint (Figure 5A,C). No differences in high dose response were observed at 24 hours after injection. The low dose response (0.3 μg) did not display any statistically significant reduction in mechanical hypersensitivity at 3 or 7 weeks post-fracture. Similarly, intraplantar administration of the CCR2 antagonist at two doses (1 and 10 μg) did not affect mechanical hypersensitivity at 3 or 7 weeks post-fracture (Figure 5B,D). An additional study testing the effects of a higher CCR2 antagonist dose (30 μg) failed to show anti-nociceptive efficacy (data not shown).

### Both intrathecal and intraplantar CcL2 induce transient mechanical hypersensitivity

A dose-dependent transient mechanical hypersensitivity was observed following intrathecal and intraplantar administration of CcL2 (Additional file 5: Figure S2A,B); measurements were taken 1, 3, 6 and 24 hours after injections. Statistical analysis identified a Dose × Time interaction in both administration routes (Intrathecal, (A): 2-way ANOVA; Dose × Time Interaction, F-value = 14.30; p < 0.0001. Intraplantar, (B): 2-way ANOVA; Dose × Time Interaction, F-value = 7.98; p < 0.0001).

Intrathecal doses of 10 and 100 ng were capable of inducing mechanical hypersensitivity 1 and 3 hours after injection with the higher dose demonstrating hypersensitivity 6 hours after injection. The higher dose induced significantly greater hypersensitivity compared to the lower doses at the 1, 3 and 6-hour timepoint (Additional file 5: Figure S2A).

Intraplantar administration of CcL2 at increased doses (100 and 500 μg) demonstrated similar profiles with increased mechanical hypersensitivity at 1, 3 and 6-hour timepoints for both low and high doses. A dose-dependent effect was also noted with the higher dose inducing significantly greater hypersensitivity at the 3 and 6-hour timepoints (Additional file 5: Figure S2B).

## Discussion

Our lack of effective treatments for CRPS may be the result, at least in part, of a lack of knowledge of the molecules and mechanisms supporting the disease. Using a hypothesis-free approach to gene discovery involving expression arrays constitutes a novel approach to understanding this condition. For our studies, we employed a previously characterized model of CRPS involving limb fracture and cast immobilization in rodents [20]. Our results showed that these animals develop behavioral signs of nociceptive sensitization in terms of weight bearing and mechanical allodynia in addition to physiological signs characteristic of clinical CRPS such as temperature changes and edema in the affected hindpaw. Furthermore, we demonstrated that the acute (3 weeks) and chronic (7 weeks) phases of CRPS in our model are accompanied by unique changes in spinal gene expression. A diverse array of biological pathways were suggested to be activated in the spinal cord tissue of fracture/cast mice, many of which currently have little appreciated relevance to pain or the manifestations of CRPS. On the other hand, one of the genes prominently up-regulated in the acute phase is CcL2, a gene found previously to be a possible common pain gene in many array studies [19]. This up-regulation was confirmed both at the mRNA and protein levels. Finally, the spinal administration of a CCR2 antagonist in CRPS animals decreased mechanical allodynia while both the spinal and peripheral administration of CcL2 itself resulted in mechanical allodynia in control mice. Our results point to a diverse, changing, and previously unappreciated complexity of genes possibly involved in the varied manifestations of CRPS. At the same time, our studies confirm that there may be at least one gene involved in CRPS that is shared with chronic neuropathic and inflammatory pain.

### Acute vs. Chronic stages of CRPS

Chronic pain is a debilitating condition affecting many aspects of the patient’s life including reduced quality of life (>50%), negative impact on relationships (29%), job loss or reduced job responsibilities (>50%), increased rates of depression (30%), and twice the likelihood of suicide while awaiting treatment [21]. Many forms of chronic pain begin with an acute injury or syndrome. For CRPS patients, a relatively acute or “warm” syndrome characterized by pain, edema and warmth often gives way to a “cold” phase in which pain persists after the resolution of the vascular changes [10]. Unfortunately, it is difficult to predict the circumstances under which acute pain will transition into a chronic state. This is partly due to our limited understanding concerning the molecular mechanisms of pain, in general, and to a lack of a clear evolution point between the acute and chronic phases, in particular.

Apart from many cases observed in children [22], CRPS often becomes a chronic clinical problem. It is possible that segregating the acute effects from the chronic ones could enable us to target the acute changes in an effort to prevent the chronification of pain. We have delineated, for the first time, the spinal transcriptomic changes in acute (3 weeks characterized by allodynia, unweighting, warmth and ede ma) and chronic (7 weeks, characterized by allodynia and unweighting without vascular changes) stages of CRPS-like changes in mice. We identified molecular pathways unique to each of the acute and chronic phases. Comparison analysis of the microarray data obtained at the two timepoints revealed the top functional pathways involved in the acute phase as: cellular movement, cancer, cardiovascular system development and function, organismal development, nutritional disease, and cell death and survival. These pathways are consistent with the early stage CRPS pathology in our model. For instance, bone fracture and acute pain both can have immense global effects on both sensory and motor function, and as such, could be associated with a certain level of cellular re-organization (involvement of functions such as cellular movement and cell death and survival) at the spinal level. Similarly, the weight loss associated with the earlier timepoints in our model could account for the involvement of pathways such as organismal development and nutritional disease. As for the more chronic phase, the top pathways were: inflammatory response, hematological system development and function, immune cell trafficking, cell-to-cell signaling and interaction, and cellular movement. These pathways parallel the chronic phase of CRPS in our model, with particular emphasis on the neuro-immune component (inflammatory response and immune-cell trafficking). The chronic pain phenotype also implies central sensitization and thus explains the involvement of pathways such as cell-to-cell signaling and interaction and cellular movement. On the other hand, pathways such as those involved in cancer are more difficult to relate to CRPS, a benign condition. Perhaps the activation of these pathways is best viewed not as evidence of transformation of cells to a malignant state, but rather reflective of profound changes in function. Despite some overlap in the transcriptomic changes between the two timepoints, significant changes in gene expression reflected in the changes in pathway analysis imply a progression or transition in the pathology of the disease, mirroring the phenotypic changes observed in our model.

Our findings complement previous microarray studies undertaken both in dorsal root ganglia (DRG) and spinal cord samples in different animal models of pain [19]. For instance, acute transcriptomic changes were reported in the zymosan model of DRG inflammation and included pathways such as: defense response, immune response, regulation of body fluid levels, osteoblast proliferation, hemopoietic or lymphoid organ development, leukocyte proliferation, neuronal generation, epithelial cell proliferation, etc. 3 days after the induction of inflammation [23]. In models of neuropathy, functional gene clusters relating to complement activation, antigen processing/presentation, neuronal axonogenesis, cell adhesion, synaptic transmission, etc. were identified [19]. While it could be argued that the type of injury (neuropathic versus inflammatory) could potentially be a more important contributing factor than the timecourse [24] of injury/pain, we suggest that both the type of insult (inflammatory versus neuropathic) and the timecourse are reflected in the transcriptional changes and may be intertwined. For instance, inflammation and neuropathy could both be linked to a multifactorial syndrome such as CRPS, and the contribution of each could change with time.

### Role of CcL2 in nociceptive processing

Similar to previous findings [25], our data shows that both the intrathecal and intraplantar administration of CcL2 result in mechanical allodynia. This is in agreement with the global role of CcL2 in nociception. The function of CcL2 in peripheral nociception, in particular at the level of the DRG and afferent neurons, has been well described. Electrophysiological studies have shown that CcL2 depolarizes DRG neurons in neuropathic animals [26,27] and sensitizes nociceptors through the activation of TRP channels and the inhibition of K+ conduction [26,28,29]. As elegant as these studies are, our own data involving the local injection of a selective CCR2 antagonist suggest that distal peripheral sites of CcL2 action are not prominent in supporting sensitization in our model.

In contrast, intrathecal injection of the same CCR2 antagonist reduced mechanical allodynia significantly, thus demonstrating that the CcL2 upregulation observed in the array and ELISA experiments was functional in our model. Unfortunately, CcL2 expression in the spinal cord remains controversial [30]. Although we did not specifically explore the question in our model, it has been shown that injured and uninjured neurons [31-33], astrocytes [34], and microglia [35] could all be potential sources for the elevated levels of CcL2. We did determine, however, that the upregulation of spinal CcL2 expression occurs in the absence of any measurable transcriptional changes in astrocyte (GFAP) and macrophage (Iba1) markers at both the 3- and 7-week time points. It is possible that CRPS is associated with microglial activation without wide-spread microglial proliferation, with activated microglia secreting a variety of pro-inflammatory cytokines and chemokines that are implicated in nociception. Though it is known that endogenous CcL2 can induce microglial activation in a mouse model of neuropathic pain [36], it is not known if this is true for the CRPS model. It would therefore be interesting to examine microglial and astrocytic responses following the fracture/cast procedure, whether these cells are responsible for some of the gene expression demonstrated by the arrays, and the responses of glial cells to the spinal administration of CcL2.

Another notable point is the timecourse of CcL2 upregulation in relation to phenotypic changes in our model. We show a transient upregulation of CcL2 mRNA and protein in the ipsilateral spinal cord at 3 weeks after fracture with the return to levels very close to control samples by 7 weeks. However, mechanical allodynia in this model persists well beyond 7 weeks, and retains its sensitivity to a CCR2 antagonist. One explanation is that CcL2 and other mediators play roles in the early cascade of events that lead to enhanced nociceptive sensitization that persists even after the resolution of the early changes. Thus at later time points even normal levels of CcL2 may support allodynia. In fact, plasma levels of CcL2 in CRPS patients were found to show no correlation with the duration of the CRPS [37], and CSF levels of CcL2 and GFAP were found to be upregulated in only 50% of patients [38], suggesting that mechanisms other than persistent chemokine activation are at play.

### CcL2/CCR2 As potential therapeutic targets

The involvement of CcL2 in pain makes it an attractive candidate for therapeutic intervention. Indeed, multiple studies have shown that antagonizing CCR2, a major receptor for CcL2, is efficacious in reversing allodynia and hyperalgesia in animal models of neuropathic pain [39,40]. As mentioned above, our data show that spinal but not local hindpaw administration of the CCR2 antagonist, RS504393, attenuates mechanical sensitivity observed in our CRPS model. These observations are distinct from a report where CcL2-induced CCR2 receptor activation was shown to occur mainly in the peripheral nervous system in a demyelination model of neuropathic pain [41]. These differences could be due to the possibility that in our CRPS model, unlike neuropathy models, CcL2 plays its primary role in the CNS. While CcL2 itself is sufficient to induce mechanical sensitivity in the periphery, this type of signaling may not be necessary to induce and/or maintain CRPS-related nociception in the effected hindpaw. This is not to say peripheral inflammatory mediators are not involved in CRPS. Similar to clinical observations [42], data from our rat tibia fracture/cast immobilization model of CRPS has shown the upregulation of many peripheral inflammatory cytokines [43,44], thus drawing attention to the peripheral mechanisms that support some chronic pain syndromes.

The CCR2 antagonist AZD2423 was recently evaluated as an analgesic in a well-designed clinical trial, but had no efficacy in patients with posttraumatic neuralgia [45]. On the other hand, additional persistent pain states remain viable targets for this or similar drugs. Alternatively, it is possible that, in humans, CcL2 does not play as strong a role or perhaps is only one of many mediators ultimately converging on the same pathways to maintain pain in CRPS. Consistent with this notion, our array analysis revealed that multiple CC chemokine family members underwent alterations in expression making a reduction in signaling through one molecule unlikely to completely eliminate the nociceptive sensitization characterizing this syndrome. Perhaps a more systems-oriented approach would be useful when targeting the treatment of complex and multifactorial pathologies such as chronic pain.

### Limitations and future directions

While this work has identified molecules and pathways hopefully expanding our understanding of acute and more chronic forms of CRPS, our approach has several caveats. Microarray probes do have inherent biases in their design due to their reliance on annotated genes. A more comprehensive approach, such as RNA sequencing, could provide a truly unbiased view [46]. In addition, the current manuscript demonstrates a nociceptive role of CcL2 in a mouse model of CRPS but it does not inform us about its source. Localizing CcL2 and/or CCR2 to a specific cell type would improve our understanding of the function of CcL2 in CRPS.

Finally, the current work examines spinal effects only while it is well known that CRPS has significant peripheral [44,47] and supraspinal [48,49] correlates. Ultimately, understanding how changes in gene expression and function at each of these sites contribute will be necessary to fully understand the syndrome.

## Conclusions

We demonstrate broad and distinct changes in gene expression in the ipsilateral cord during the acute and chronic phases of “CRPS” in a tibia fracture/immobilization model in mice. These data allow the comparison on a broad-based molecular level with other models of chronic pain, and support the generation of novel hypotheses regarding mechanisms supporting acute and more chronic forms of CRPS. One particular gene of interest, CcL2, was found to be upregulated in the acute phase of CRPS and was pharmacologically shown to be associated with nociceptive thresholds at both acute and more chronic time points. Thus, there exists at least one functional example of a gene functioning in this very unique pain model in ways similar to those observed in models of inflammatory and neuropathic pain.

## Methods

### Animals

Male C57/B6J mice aged 12–14 weeks were purchased from a commercial supplier (Jackson Labs, Sacramento, CA) and were allowed to habituate to the animal facility for a minimum of 10 days prior to the experiments. Mice were housed in groups of 4 in a cage on a 12-hr light/dark cycle and an ambient temperature of 22 ± 1°C, with food and tap water available *ad libitum*. All animal procedures were approved by the Veterans Affairs Palo Alto Health Care System Institutional Animal Care and Use Committee (Palo Alto, CA) and followed the animal subjects guidelines of the International Association for the Study of Pain published in PAIN, 16 (1983) 109–110.

### Limb fracture and cast immobilization

Mice were anesthetized with 1.5% Isoflurane in air and received a distal tibial fracture. Immediately following fracture and while still under anesthetic, a cast was placed around the injured hindpaw so as to encase it entirely [15]. To ensure the proper and consistent placement of the cast, it was elongated to wrap about the abdomen of the animal. After fracture and casting, the mice were given subcutaneous Buprenorphine (0.05 mg/kg) and Baytril (5 mg/kg) for the next three days, as well as normal saline (1.5 ml once) for post-operative analgesia, prevention of infection and dehydration. Mice were inspected daily to ensure the cast was positioned properly through the 3-week period of cast immobilization. Mice were provided with chow *ad libitum*; diet gels were also made available on the cage floor for mice having undergone surgery. At 3 weeks after surgery, the mice were anesthetized and the casts were removed using pliers.

### Timecourse

Behavioral and physiological measures were taken on the injured and contralateral hindpaw 3, 5 and 7 weeks after fracture; the 3-week timepoint was 24 hours after cast removal. Tissue collected at the 3 and 7-week timepoints was used for the molecular studies (microarray, qPCR, ELISA).

### Physiological measures

#### Hindpaw temperature

The temperature of the hindpaw was measured using a fine wire thermocouple (Omega) applied to the paw skin, as described previously [50]. The investigator held the wire using an insulating Styrofoam block. Three sites were tested over the dorsum of the hindpaw: the space between the first and second metatarsals (medial), the second and third metatarsals (central), and the fourth and fifth metatarsals (lateral). After a site was tested in one hindpaw the same site was immediately tested in the contralateral hindpaw. The testing protocol was medial dorsum right then left, central dorsum right then left, lateral dorsum right then left, medial dorsum left then right, central dorsum left then right, and lateral dorsum left then right. The six measurements for each hindpaw were averaged for the mean temperature.

#### Hindpaw volume

A laser sensor technique was used to determine the dorsal–ventral thickness of the hindpaw, as we have previously described [50]. The measurement sensor device used in these experiments (Limab) has a measurement range of 200 mm with a 0.01 mm resolution.

### Behavioral testing

#### Mechanical hypersensitivity

Calibrated monofilaments (Stoelting Co., Wood Dale, IL) were applied to the plantar surface of the hindpaw and the 50% threshold to withdraw (grams) was calculated as previously described [51].

#### Hindpaw unweighting

An incapacitance device (IITC Inc.) was used to measure hindpaw unweighting. The mice were manually held in a vertical position over the apparatus with the hindpaws resting on separate metal scale plates and the entire weight of the mouse was supported on the hindpaws. The duration of each measurement was 6 s and 6 consecutive measurements were taken at 10s intervals. All 6 readings were averaged to calculate the bilateral hindpaw weight-bearing values.

### Microarray analysis

Microarray analysis was performed in the L4/L5 region of the ipsilateral spinal cord at 3 and 7 weeks in CRPS (n = 3/group; tissue from 2 animals combined for each sample) and age-matched control mice. Microarray profiling was performed using an Agilent Whole Mouse Genome Microarray (44 k arrays) at the Stanford Functional Genomics Facility. Results were imported into R using the Bioconductor package for microarray analysis where RMA normalization was performed and filtering of absent/present labels performed. Statistical analysis of the 3- and 7-week timepoint compared to the control timepoint was then performed using Students *t* test. False Discovery Rate (FDR) was used to adjust p-values to take into account multiple comparison effects and significance was considered reached at an absolute fold change of 1.5 and a FDR-adjusted p-value of less than 0.05.

### Pathway analysis

Ingenuity Software was used to perform whole pathway analysis in the identification of affected networks and their relationship to each other based on the differential expression between control, 3- and 7-week CRPS mice. Briefly, our data set containing gene identifiers and corresponding expression values was uploaded into the application. Each identifier was mapped to its corresponding object in the Ingenuity® Knowledge Base. Differentially expressed genes, called network eligible molecules, were overlaid onto a global molecular network developed from information contained in the Ingenuity Knowledge Base. Networks of Network Eligible Molecules were then algorithmically generated based on their connectivity. The pathway presented was chosen from following candidate gene validation. Right-tailed Fisher’s exact test was used to calculate a p-value determining the probability that the pathway is due to chance alone.

### RNA isolation and quantitative real-time polymerase chain reaction (PCR)

Mice were first euthanized by carbon dioxide asphyxiation and spinal cord tissue was harvested by extrusion. Ipsilateral L4/L5 lumbar spinal cord segments were dissected on a chilled surface. Dissected tissue was then quick-frozen in liquid nitrogen and stored at −80°C until required for analysis. Total RNA was isolated from spinal cord using the RNeasy Mini Kit (Qiagen) according to the manufacturer's instructions. The purity and concentration were determined spectrophotometrically. The cDNA was subsequently synthesized using an iScript cDNA Synthesis Kit (Bio-Rad Laboratories). Real time PCR reactions were conducted using the SYBR Green PCR master mix (Applied Biosystems) and performed on the ABI 7900HT sequencing detection system (Applied Biosystems). The data from real time PCR experiments were analyzed by the comparative CT method as described in the manufacturer’s manual.

### Enzyme-linked immunosorbent assay (ELISA)

Mice were first euthanized by carbon dioxide asphyxiation and spinal cord tissue was harvested by extrusion. Lumbar spinal cord segments were dissected on a chilled surface. Dissected tissue was then quick-frozen in liquid nitrogen and stored at −80°C until required for analysis. Mouse lumbar spinal cords were homogenized in ice cold 0.9% NaCl containing a cocktail of protease inhibitors (Roche Applied Science) and centrifuged at 12,000G for 10 min at 4°C. Supernatant fractions were then frozen at −80°C until use. An aliquot was subjected to protein assay (Bio-Rad) to normalize mediator levels. CcL2 levels were assayed in duplicate by using mouse CcL2 ELISA kit (R&D Systems) according to the manufacturer’s instructions.

### Pharmacology

#### CcL2 administration

Recombinant full length CcL2 (ab9901, Abcam) was reconstituted in sterile saline. CcL2 or saline were administered intrathecally (10 or 100 ng in 5 μL; n = 8/group) or through intraplantar injections (100 or 500 μg in 10 μl).

#### RS5044393 administration

The CCR2 antagonist, RS504393 (Santa Cruz Biotech, CA) was diluted in sterile saline containing DMSO (<1 %). RS504393 or vehicle (saline with 1% DMSO) were administered intrathecally (0.3 or 3 μg in 5 μL) or through intraplantar injections (1 or 10 μg in 10 μl).

### Statistical analysis

All data are expressed as mean ± SEM. Analysis of repeated parametric measures was accomplished using a one-way analysis of variance followed post-hoc Dunnett or Bonferroni testing or a two-way analysis of variance followed by Bonferroni testing. For simple comparisons of two groups, a two-tailed Student t-test was employed. Welch’s correction was used when the assumption of equal variances was not met. Significance was set at p < 0.05. (Prism 5; GraphPad Software, La Jolla, CA).

## Abbreviations

CcL2: Chemokine (C-C motif) Ligand 2; CCR2: C-C Chemokine Receptor 2; CNS: Central nervous system; CRPS: Complex Regional Pain Syndrome; DRG: Dorsal root ganglion; ELISA: Enzyme-linked immunosorbent assay; IPA: Ingenuity Pathway Analysis; SC: Spinal Cord; qPCR: Quantitative Polymerase Chain Reaction.

## Competing interests

The authors do not have financial or other relationships that might lead to conflict of interest.

## Authors’ contributions

JJ: Overall experimental design, microarray analysis, behavioral testing, writing of the manuscript. MT: Ingenuity pathway analysis, behavioral testing, writing of the manuscript. TG: Behavioral testing, tissue collection. WL: Generation of CRPS model animals, tissue collection. XS: ELISA, RNA extraction, qPCR analysis. MZ: Array quality control and interpretation, Ingenuity pathway analysis. GP: Supervision of array analysis. WSK: Co-PI of project, interpretation of results. JDC: Co-PI of project, overall supervision of experiments. All authors read and approved the final manuscript.

## Supplementary Material

Additional file 1: Table S1 Microarray results in ipsilateral spinal cord 3 weeks post fracture. Click here for file

Additional file 2: Table S2 Microarray results in ipsilateral spinal cord 7 weeks post fracture. Click here for file

Additional file 3: Table S3 Comparative analysis of functional pathways regulated 3 and 7 weeks post fracture. Shaded cells indicate the 3-week timepoint. Click here for file

Additional file 4: Figure S1 Validation of transcript mRNA expression. qPCR validation of upregulated **(A)**, downregulated **(B)**, and unchanged **(C)** transcripts in the ipsilateral spinal cord 3 and 7 weeks after fracture. The dotted line indicates control measures. * p<0.05. n=4/group. Errors bars=S.E.M. Click here for file

Additional file 5: Figure S2 Induction of mechanical allodynia following the intrathecal and intraplantar administration of CcL2. Both intrathecal **(A)** and intraplantar **(B)** administration of CcL2 resulted in decreased mechanical thresholds compared to vehicle-treated mice. *** p<0.001. n=7-8/group. Errors bars=S.E.M. Click here for file
