# Supplementary material for: Acute and chronic phases of complex regional pain syndrome in mice are accompanied by distinct transcriptional changes in the spinal cord
Source: Mol Pain. 2013 Aug 8;9:40. doi: 10.1186/1744-8069-9-40 (PMC3751593; doi:10.1186/1744-8069-9-40)
Supplement: Additional file 1: Table S1 — Microarray results in ipsilateral spinal cord 3 weeks post fracture. [file 1744-8069-9-40-S1.pdf]

SYMBOL	DESCRIPTION	Fold Change
**Scgb3a1**	secretoglobin, family 3A, member 1	9.21
**Sprr1a**	small proline-rich protein 1A	8.88
**Atf3**	activating transcription factor 3	4.18
**Ccl2**	chemokine (C-C motif) ligand 2	4.17
**Ccl7**	chemokine (C-C motif) ligand 7	4.01
**Gal**	galanin	3.86
**4933402N22Rik**	RIKEN cDNA 4933402N22 gene	3.69
**Pdyn**	prodynorphin	3.01
**Srsf2**	serine/arginine-rich splicing factor 2	2.41
**Egr4**	early growth response 4	2.38
**Wdfy1**	WD repeat and FYVE domain containing 1	2.28
**Mustn1**	musculoskeletal, embryonic nuclear protein 1	2.19
**Adcyap1**	adenylate cyclase activating polypeptide 1	2.07
**Ogfod1**	2-oxoglutarate and iron-dependent oxygenase domain containing 1	2.02
**Npy**	neuropeptide Y	2.02
**F2rl2**	coagulation factor II (thrombin) receptor-like 2	1.97
**Cd34**	CD34 antigen	1.91
**Cbx6**	chromobox homolog 6	1.91
**Cartpt**	CART prepropeptide	1.90
**Socs3**	suppressor of cytokine signaling 3	1.89
**Adamtsl2**	ADAMTS-like 2	1.87
**Speer5-ps1**	spermatogenesis associated glutamate (E)-rich protein 5, pseudogene 1	1.86
**Inhba**	inhibin beta-A	1.82
**Ddx5**	DEAD (Asp-Glu-Ala-Asp) box polypeptide 5	1.79
**Ctla2b**	cytotoxic T lymphocyte-associated protein 2 beta	1.78
**Speer4d**	spermatogenesis associated glutamate (E)-rich protein 4d	1.78
**Serpinb11**	serine (or cysteine) peptidase inhibitor, clade B (ovalbumin), member 11	1.74
**6330578E17Rik**	RIKEN cDNA 6330578E17 gene	1.71
**Slc12a6**	solute carrier family 12, member 6	1.71
**Dennd4a**	DENN/MADD domain containing 4A	1.71
**Hoxd10**	homeobox D10	1.70

**Gpr4**	G protein-coupled receptor 4	1.70
**Cebpd**	CCAAT/enhancer binding protein (C/EBP), delta	1.70
**Cog6**	component of oligomeric golgi complex 6	1.69
**Btg2**	B-cell translocation gene 2, anti-proliferative	1.68
**Arl6ip6**	ADP-ribosylation factor-like 6 interacting protein 6	1.68
**Cttn**	cortactin	1.67
**Rims1**	regulating synaptic membrane exocytosis 1	1.66
**Spint1**	serine protease inhibitor, Kunitz type 1	1.66
**Tspyl5**	testis-specific protein, Y-encoded-like 5	1.65
**Suv39h2**	suppressor of variegation 3-9 homolog 2 (Drosophila)	1.64
**Akap12**	A kinase (PRKA) anchor protein (gravin) 12	1.64
**Klf2**	Kruppel-like factor 2 (lung)	1.64
**Ank3**	ankyrin 3, epithelial	1.63
**Abhd15**	abhydrolase domain containing 15	1.63
**Eif2s3x**	eukaryotic translation initiation factor 2, subunit 3, structural gene X-linked	1.62
**Pde10a**	phosphodiesterase 10A	1.61
**Hltf**	helicase-like transcription factor	1.61
**Auts2**	autism susceptibility candidate 2	1.61
**Ets1**	E26 avian leukemia oncogene 1, 5' domain	1.61
**Mgp**	matrix Gla protein	1.61
**Sbno2**	strawberry notch homolog 2 (Drosophila)	1.59
**Rbm17**	RNA binding motif protein 17	1.59
**Parva**	parvin, alpha	1.59
**Pcsk1**	proprotein convertase subtilisin/kexin type 1	1.59
**Rpsa**	ribosomal protein SA	1.59
**Darc**	Duffy blood group, chemokine receptor	1.58
**Son**	Son DNA binding protein	1.57
**Tgm2**	transglutaminase 2, C polypeptide	1.57
**Reep2**	receptor accessory protein 2	1.57
**6-Sep**	septin 6	1.56
**Eef2k**	eukaryotic elongation factor-2 kinase	1.55
**Jazf1**	JAZF zinc finger 1	1.54

**Arg1**	arginase, liver	1.54
**Pknox1**	Pbx/knotted 1 homeobox	1.54
**Meg3**	maternally expressed 3	1.53
**Trank1**	tetratricopeptide repeat and ankyrin repeat containing 1	1.53
**Atp2a2**	ATPase, Ca++ transporting, cardiac muscle, slow twitch 2	1.53
**Dusp2**	dual specificity phosphatase 2	1.52
**Lpcat1**	lysophosphatidylcholine acyltransferase 1	1.52
**Slc35a5**	solute carrier family 35, member A5	1.52
**Acta2**	actin, alpha 2, smooth muscle, aorta	1.52
**Ier3**	immediate early response 3	1.52
**Slc30a7**	solute carrier family 30 (zinc transporter), member 7	1.51
**Psd**	pleckstrin and Sec7 domain containing	1.51
**Ptprn**	protein tyrosine phosphatase, receptor type, N	1.51
**8430419L09Rik**	RIKEN cDNA 8430419L09 gene	1.51
**Lhfp**	lipoma HMGIC fusion partner	1.51
**Fzd2**	frizzled homolog 2 (Drosophila)	1.51
**C030046I01Rik**	RIKEN cDNA C030046I01 gene	1.51
**2010005H15Rik**	RIKEN cDNA 2010005H15 gene	1.51
**Fosl1**	fos-like antigen 1	1.50
**1700001O22Rik**	RIKEN cDNA 1700001O22 gene	1.50
**Jak3**	Janus kinase 3	1.50
**Gprc5b**	G protein-coupled receptor, family C, group 5, member B	0.13
**Loxl2**	lysyl oxidase-like 2	0.29
**Slc36a2**	solute carrier family 36 (proton/amino acid symporter), member 2	0.40
**Ly6d**	lymphocyte antigen 6 complex, locus D	0.43
**Mapk8**	mitogen-activated protein kinase 8	0.45
**Ccl5**	chemokine (C-C motif) ligand 5	0.46
**Zswim6**	zinc finger, SWIM domain containing 6	0.46
**Cadm3**	cell adhesion molecule 3	0.48
**1700030C10Rik**	RIKEN cDNA 1700030C10 gene	0.48
**Clec7a**	C-type lectin domain family 7, member a	0.48

**Islr**	immunoglobulin superfamily containing leucine-rich repeat	0.51
**Alox12**	arachidonate 12-lipoxygenase	0.52
**Slc34a3**	solute carrier family 34 (sodium phosphate), member 3	0.53
**Serpind1**	serine (or cysteine) peptidase inhibitor, clade D, member 1	0.53
**Prss23**	protease, serine, 23	0.55
**Nkain1**	Na+/K+ transporting ATPase interacting 1	0.55
**Cadm2**	cell adhesion molecule 2	0.55
**Spn**	sialophorin	0.55
**Rhd**	Rh blood group, D antigen	0.55
**Myoc**	myocilin	0.56
**Nostrin**	nitric oxide synthase trafficker	0.56
**Ide**	insulin degrading enzyme	0.56
**Akap2**	A kinase (PRKA) anchor protein 2	0.56
**Hist1h2ak**	histone cluster 1, H2ak	0.57
**Ccrl1**	chemokine (C-C motif) receptor-like 1	0.57
**Tnfaip2**	tumor necrosis factor, alpha-induced protein 2	0.58
**Loxl1**	lysyl oxidase-like 1	0.58
**Lmnb1**	lamin B1	0.58
**Ramp1**	receptor (calcitonin) activity modifying protein 1	0.58
**Myh8**	myosin, heavy polypeptide 8, skeletal muscle, perinatal	0.59
**Fzd10**	frizzled homolog 10 (Drosophila)	0.59
**Exoc6b**	exocyst complex component 6B	0.59
**Pllp**	plasma membrane proteolipid	0.60
**Gab3**	growth factor receptor bound protein 2-associated protein 3	0.60
**Expi**	extracellular proteinase inhibitor	0.60
**Hdc**	histidine decarboxylase	0.60
**Nhedc2**	Na+/H+ exchanger domain containing 2	0.60
**Igfbp4**	insulin-like growth factor binding protein 4	0.61
**Cenpe**	centromere protein E	0.61
**Ptger3**	prostaglandin E receptor 3 (subtype EP3)	0.61
**P2ry12**	purinergic receptor P2Y, G-protein coupled 12	0.62
**Txlnb**	taxilin beta	0.62

**Ltb**	lymphotoxin B	0.62
**Prom1**	prominin 1	0.62
**Adamts2**	a disintegrin-like and metallopeptidase (reprolysin type) with thrombospondin type 1 motif, 2	0.62
**Nufip2**	nuclear fragile X mental retardation protein interacting protein 2	0.62
**Cnksr3**	Cnksr family member 3	0.63
**Sdad1**	SDA1 domain containing 1	0.63
**Pygl**	liver glycogen phosphorylase	0.63
**2210012G02Rik**	RIKEN cDNA 2210012G02 gene	0.64
**Pcdh12**	protocadherin 12	0.64
**Ugt8a**	UDP galactosyltransferase 8A	0.64
**Tprkb**	Tp53rk binding protein	0.65
**Cmya5**	cardiomyopathy associated 5	0.65
**Enpp1**	ectonucleotide pyrophosphatase/phosphodiesterase 1	0.65
**Rnf13**	ring finger protein 13	0.65
**Icam4**	intercellular adhesion molecule 4, Landsteiner-Wiener blood group	0.65
**Pgm5**	phosphoglucomutase 5	0.65
**Nckap5**	NCK-associated protein 5	0.65
**Agtr2**	angiotensin II receptor, type 2	0.65
**Itgal**	integrin alpha L	0.65
**Bace2**	beta-site APP-cleaving enzyme 2	0.65
**Hmcn1**	hemicentin 1	0.65
**S100a6**	S100 calcium binding protein A6 (calcyclin)	0.65
**Fam98b**	family with sequence similarity 98, member B	0.65
**Slc26a10**	solute carrier family 26, member 10	0.65
**Sned1**	sushi, nidogen and EGF-like domains 1	0.66
**Tecrl**	trans-2,3-enoyl-CoA reductase-like	0.66
**Ssr1**	signal sequence receptor, alpha	0.66
**Lrba**	LPS-responsive beige-like anchor	0.66
**Hhip**	Hedgehog-interacting protein	0.66
**1700009P17Rik**	RIKEN cDNA 1700009P17 gene	0.66
**A530054K11Rik**	RIKEN cDNA A530054K11 gene	0.66

**Pgam2**	phosphoglycerate mutase 2	0.66
**Aspa**	aspartoacylase	0.66
