# Supplementary material for: Acute and chronic phases of complex regional pain syndrome in mice are accompanied by distinct transcriptional changes in the spinal cord
Source: Mol Pain. 2013 Aug 8;9:40. doi: 10.1186/1744-8069-9-40 (PMC3751593; doi:10.1186/1744-8069-9-40)
Supplement: Additional file 2: Table S2 — Microarray results in ipsilateral spinal cord 7 weeks post fracture. [file 1744-8069-9-40-S2.pdf]

Symbol	Description	Fold change
**Ogg1**	8-oxoguanine DNA-glycosylase 1	4.12
**Fam13c**	family with sequence similarity 13, member C	2.47
**Cd274**	CD274 antigen	2.03
**Man2a2**	mannosidase 2, alpha 2	1.92
**Hist1h1e**	histone cluster 1, H1e	1.91
**Zfp445**	zinc finger protein 445	1.79
**Olfr921**	olfactory receptor 921	1.76
**Elavl3**	ELAV (embryonic lethal, abnormal vision, Drosophila)-like 3 (Hu antigen C)	1.76
**Glyctk**	glycerate kinase	1.74
**Frmd5**	FERM domain containing 5	1.72
**Esr1**	estrogen receptor 1 (alpha)	1.72
**Npnt**	nephronectin	1.72
**Gsta3**	glutathione S-transferase, alpha 3	1.68
**Actr8**	ARP8 actin-related protein 8 homolog (S. cerevisiae)	1.66
**Tcf15**	transcription factor 15	1.65
**Cyld**	cylindromatosis (turban tumor syndrome)	1.65
**Ppp4r1l-ps**	protein phosphatase 4, regulatory subunit 1-like, pseudogene	1.64
**Cntn5**	contactin 5	1.62
**Kif3b**	kinesin family member 3B	1.62
**A830010M20Rik**	RIKEN cDNA A830010M20 gene	1.61
**Cyr61**	cysteine rich protein 61	1.61
**Rapgef6**	Rap guanine nucleotide exchange factor (GEF) 6	1.60
**Serpine2**	serine (or cysteine) peptidase inhibitor, clade E, member 2	1.60
**2410066E13Rik**	RIKEN cDNA 2410066E13 gene	1.59
**Vprbp**	Vpr (HIV-1) binding protein	1.59
**Grap**	GRB2-related adaptor protein	1.59
**Stk16**	serine/threonine kinase 16	1.57
**Ets1**	E26 avian leukemia oncogene 1, 5' domain	1.57
**Luzp1**	leucine zipper protein 1	1.57
**Srp54a**	signal recognition particle 54A	1.56
**Crebl2**	cAMP responsive element binding protein-like 2	1.56
**Snx30**	sorting nexin family member 30	1.55
**Sec23ip**	Sec23 interacting protein	1.55
**Msto1**	misato homolog 1 (Drosophila)	1.55
**Col24a1**	collagen, type XXIV, alpha 1	1.54
**Brd3**	bromodomain containing 3	1.54
**Wnt11**	wingless-related MMTV integration site 11	1.54
**Ppp1r9a**	protein phosphatase 1, regulatory (inhibitor) subunit 9A	1.52
**Zfp169**	zinc finger protein 169	1.51
**Anp32e**	acidic (leucine-rich) nuclear phosphoprotein 32 family, member E	1.51
**Fam134b**	family with sequence similarity 134, member B	1.51
**Xpo4**	exportin 4	1.51
**Dscam**	Down syndrome cell adhesion molecule	1.50

**Ccnl1**	cyclin L1	1.50
**Madd**	MAP-kinase activating death domain	1.50
**Retnlg**	resistin like gamma	0.12
**Lrg1**	leucine-rich alpha-2-glycoprotein 1	0.20
**Chad**	chondroadherin	0.41
**Bst1**	bone marrow stromal cell antigen 1	0.42
**Rac2**	RAS-related C3 botulinum substrate 2	0.43
**Tspo2**	translocator protein 2	0.46
**Olfr536**	olfactory receptor 536	0.47
**Vwf**	Von Willebrand factor homolog	0.48
**4930579J09Rik**	RIKEN cDNA 4930579J09 gene	0.48
**Cd52**	CD52 antigen	0.49
**Arhgdib**	Rho, GDP dissociation inhibitor (GDI) beta	0.49
**Cadm3**	cell adhesion molecule 3	0.49
**Celf3**	VCUGBP, Elav-like family member 3	0.49
**Ifi30**	interferon gamma inducible protein 30	0.50
**F9**	coagulation factor IX	0.50
**Cst7**	cystatin F (leukocystatin)	0.51
**Mepe**	matrix extracellular phosphoglycoprotein with ASARM motif (bone)	0.54
**Ttll6**	tubulin tyrosine ligase-like family, member 6	0.54
**Ifitm3**	interferon induced transmembrane protein 3	0.55
**Ccdc33**	coiled-coil domain containing 33	0.56
**Csf2rb**	colony stimulating factor 2 receptor, beta, low-affinity (granulocyte-macrophage)	0.56
**Ubl7**	ubiquitin-like 7 (bone marrow stromal cell-derived)	0.57
**BC026782**	cDNA sequence BC026782	0.57
**Ccl9**	chemokine (C-C motif) ligand 9	0.58
**Arl10**	ADP-ribosylation factor-like 10	0.58
**Nnat**	neuronatin	0.59
**Rnasek**	ribonuclease, RNase K	0.59
**Tgfb1**	transforming growth factor, beta 1	0.59
**Mt3**	metallothionein 3	0.59
**Ptprcap**	protein tyrosine phosphatase, receptor type, C polypeptide-associated protein	0.60
**Mocs1**	molybdenum cofactor synthesis 1	0.60
**Atn1**	atrophin 1	0.61
**H1fx**	H1 histone family, member X	0.61
**Gpihbp1**	GPI-anchored HDL-binding protein 1	0.61
**Lyl1**	lymphoblastomic leukemia 1	0.61
**Lrrc25**	leucine rich repeat containing 25	0.61
**Hc**	hemolytic complement	0.62
**Ctxn1**	cortexin 1	0.62
**Rarres2**	retinoic acid receptor responder (tazarotene induced) 2	0.62
**Olfml3**	olfactomedin-like 3	0.62

**Ly6a**	lymphocyte antigen 6 complex, locus A	0.62
**Cyba**	cytochrome b-245, alpha polypeptide	0.62
**1700012P22Rik**	RIKEN cDNA 1700012P22 gene	0.62
**Srebf2**	sterol regulatory element binding factor 2	0.63
**Trp73**	transformation related protein 73	0.63
**Pycard**	PYD and CARD domain containing	0.63
**Ankrd37**	ankyrin repeat domain 37	0.63
**Ccl6**	chemokine (C-C motif) ligand 6	0.63
**2210012G02Rik**	RIKEN cDNA 2210012G02 gene	0.63
**Dkkl1**	dickkopf-like 1	0.63
**Sncb**	synuclein, beta	0.63
**Zfp36l1**	zinc finger protein 36, C3H type-like 1	0.64
**Fchsd1**	FCH and double SH3 domains 1	0.64
**Xkr5**	X Kell blood group precursor-related family, member 5	0.64
**Plod2**	procollagen lysine, 2-oxoglutarate 5-dioxygenase 2	0.64
**Myrip**	myosin VIIA and Rab interacting protein	0.64
**Khsrp**	KH-type splicing regulatory protein	0.64
**Ikzf2**	IKAROS family zinc finger 2	0.64
**Tcl1b4**	T-cell leukemia/lymphoma 1B, 4	0.65
**Tst**	thiosulfate sulfurtransferase, mitochondrial	0.65
**Lrrc3b**	leucine rich repeat containing 3B	0.65
**Osmr**	oncostatin M receptor	0.65
**Zfp628**	zinc finger protein 628	0.65
**F2r**	coagulation factor II (thrombin) receptor	0.65
**Ankib1**	ankyrin repeat and IBR domain containing 1	0.66
**Podnl1**	podocan-like 1	0.66
**1700007G11Rik**	RIKEN cDNA 1700007G11 gene	0.66
**Adprhl1**	ADP-ribosylhydrolase like 1	0.66
**Olfr1143**	olfactory receptor 1143	0.66
**Was**	Wiskott-Aldrich syndrome homolog (human)	0.66
**Man2a1**	mannosidase 2, alpha 1	0.66
**Ccdc40**	coiled-coil domain containing 40	0.66
**Alppl2**	alkaline phosphatase, placental-like 2	0.66
**Hrg**	histidine-rich glycoprotein	0.66
**Chia**	chitinase, acidic	0.66
**Itgb2**	integrin beta 2	0.66
**Pelp1**	proline, glutamic acid and leucine rich protein 1	0.67
