# Supplementary material for: Acute and chronic phases of complex regional pain syndrome in mice are accompanied by distinct transcriptional changes in the spinal cord
Source: Mol Pain. 2013 Aug 8;9:40. doi: 10.1186/1744-8069-9-40 (PMC3751593; doi:10.1186/1744-8069-9-40)
Supplement: Additional file 3: Table S3 — Comparative analysis of functional pathways regulated 3 and 7 weeks post fracture. Shaded cells indicate the 3-week timepoint. [file 1744-8069-9-40-S3.pdf]

Category	p-value	Molecules
Cellular Movement	2.33E-07-7.39E-03	LY6D,SOCS3,IGFBP4,SPN,PTGER3,LTB,ALOX12,CCL5,LMNB1,HOXD10,SERPIND1,TGM2,CCL13,MGP,CCRL1,FOSL1,KLF2,DARC,SLC12A6,NPY,ETS1,PARVA,ATF3,CARTPT,SCGB3A1,HDC,MAPK8,RAMP1,ITGAL,INHBA,CLEC7A,MYOC,CCL7,CEBPD,BTG2,ADCYAP1,JAK3,CTTN,CD34,AGTR2,RPSA,ARG1
	6.34E-06-9.03E-03	RAC2,Ly6a,RARRES2,F2R,TP73,PYCARD,HRG,CELF3,TGFB1,CD274,OSMR,Ccl6,ETS1,VWF,MAN2A1,C5,ARHGDIB,SERPINE2,Ccl9,CSF2RB,ITGB2,CYLD,CYR61,ESR1,PPP1R9A,WNT11
Cancer	3.5E-07-8.19E-03	SRSF2,SOCS3,CADM3,DENND4A,KIAA1467,SLC36A2,LTB,ATP2A2,ADAMTS2,HOXD10,SDAD1,DDX5,GAL,GPR4,MGP,TNFAIP2,FOSL1,PCDH12,TXLNB,KLF2,ETS1,LRBA,SON,ATF3,SLC9B2,PDE10A,EGR4,TSPYL5,IER3,HHIP,PLLP,INHBA,ISLR,PCSK1,HMCN1,PRSS23,CEBPD,LOXL1,BTG2,PGM5,RIMS1,RHCE/RHD,ADCYAP1,CD34,CTTN,AGTR2,LY6D,AKAP12,ANK3,IGFBP4,ZNF208/ZNF729,S100A6,F2RL2,PKNOX1,SPN,PTGER3,MYH8,ALOX12,CCL5,PROM1,LOXL2,DUSP2,SERPIND1,TGM2,GPRC5B,CCL13,CCRL1,EEF2K,NUFIP2,SLC12A6,RBM17,PARVA,ENPP1,HDC,MAPK8,UGT8,RAMP1,ITGAL,ASPA,MYOC,CCL7,PSD,LPCAT1,JAK3,HLTF
	4.08E-04-9.13E-03	RAC2,RARRES2,F2R,TP73,PYCARD,HRG,NPNT,CD52,SRP54,IFITM2,IKZF2,TGFB1,CYBA,CD274,OSMR,ETS1,VWF,C5,OGG1,SERPINE2,ITGB2,CSF2RB,F9,CST7,CYLD,CYR61,ESR1,WNT11
Cardiovascular System Development and Function	1.56E-06-7.39E-03	3,CARTPT,MAPK8,RAMP1,IER3,Meg3,NOSTRIN,INHBA,SPINT1,PCSK1,CCL7,CTTN,CD34,AGTR2,ARG1
	1.52E-04-7.32E-03	ETS1,RAC2,F2R,TP73,ZFP36L1,VWF,C5,HRG,CSF2RB,ITGB2,TGFB1,CYBA,LYL1,F9,CD274,CYLD,CYR61,ESR1,WNT11,LUZP1
Organismal Development	1.56E-06-7.25E-03	AKAP12,IGFBP4,SOCS3,PTGER3,LTB,ALOX12,CCL5,ATP2A2,LMNB1,HOXD10,ADAMTS2,SERPIND1,TGM2,SLC30A7,CCL13,GAL,MGP,FOSL1,KLF2,DARC,ETS1,NPY,ENPP1,ATF3,CARTPT,HDC,MAPK8,HHIP,Meg3,INHBA,NOSTRIN,SPINT1,SLC34A3,PCSK1,CCL7,CEBPD,LOXL1,RIMS1,ADCYAP1,JAK3,CD34,AGTR2,ARG1
	1.52E-04-5.95E-03	ETS1,RAC2,TCF15,HIST1H1E,F2R,ZFP36L1,C5,MAN2A1,HRG,CSF2RB,ITGB2,TGFB1,CYBA,CYLD,MAN2A2,CYR61,ESR1,WNT11,LUZP1
Nutritional Disease	4.94E-06-7.39E-03	NPY,SOCS3,ENPP1,ATF3,PTGER3,CARTPT,HDC,MAPK8,LTB,CCL5,INHBA,TGM2,SLC34A3,PCSK1,CCL13,SLC30A7,GAL,ADCYAP1,AGTR2
	1.81E-03-5.28E-03	TGFB1,TP73,CD274,ESR1
Cell Death and Survival	1.1E-05-7.96E-03	CSTA,SRSF2,SOCS3,DENND4A,LTB,OGFOD1,IDE,LMNB1,ATP2A2,DDX5,GAL,MGP,FOSL1,FZD2,KLF2,NPY,ETS1,SON,ATF3,PDYN,SCGB3A1,EGR4,IER3,INHBA,CEBPD,BTG2,ADCYAP1,CTTN,AGTR2,RPSA,ARG1,AKAP12,IGFBP4,S100A6,SPN,PTGER3,ALOX12,CCL5,LOXL2,DUSP2,TGM2,CCL13,EEF2K,RBM17,PARVA,MAPK8,UGT8,ITGAL,Meg3,SPINT1,Sprr1a,CCL7,JAK3
	1.06E-04-8.26E-03	CREBL2,RAC2,Ly6a,F2R,TCF15,PYCARD,TP73,VPRBP,ZFP36L1,HRG,NPNT,MADD,IKZF2,TGFB1,CYBA,LYL1,Gsta3,CD274,SNCB,Ccl6,ATN1,ETS1,PTPRCAP,PELP1,FAM134B,MT3,C5,MAN2A1,OGG1,Ccl9,SERPINE2,ITGB2,CSF2RB,SREBF2,CYLD,CYR61,ESR1,WNT11,PPP1R9A
Nucleic Acid Metabolism	1.26E-05-7.96E-03	AKAP12,NPY,SOCS3,ENPP1,PTGER3,PDE10A,RAMP1,CCL5,IDE,ATP2A2,INHBA,MYOC,GPR4,GAL,P2RY12,ADCYAP1,HLTF,AGTR2
	2.07E-03-2.07E-03	RAC2,ARHGDIB
Small Molecule Biochemistry	1.26E-05-7.96E-03	AKAP12,IGFBP4,SOCS3,PTGER3,ALOX12,IDE,CCL5,ATP2A2,TGM2,CCL13,GPR4,GAL,FOSL1,PTPRN,NPY,JAZF1,ENPP1,PDE10A,CARTPT,PDYN,HDC,MAPK8,PGAM2,UGT8,PYGL,RAMP1,ITGAL,INHBA,ASPA,SLC34A3,CLEC7A,MYOC,PCSK1,LPCAT1,CEBPD,P2RY12,ADCYAP1,CD34,HLTF,AGTR2,RPSA,ARG1
	1.1E-04-5.95E-03	ETS1,CSF2RB,RAC2,RARRES2,F2R,TGFB1,CYBA,MAN2A1,C5,CHIA,ESR1,ARHGDIB
Cell-To-Cell Signaling and Interaction	1.6E-05-7.39E-03	TPT,PDYN,HDC,MAPK8,RAMP1,ITGAL,INHBA,CLEC7A,MYOC,PCSK1,CCL7,P2RY12,SBNO2,JAK3,RHCE/RHD,ADCYAP1,CTTN,CD34,AGTR2,RPSA
	1.87E-06-9.01E-03	others),RARRES2,F2R,PYCARD,TP73,RAPGEF6,MEPE,HRG,CD52,TGFB1,CD274,Ccl6,ETS1,VWF,C5,MAN2A1,SERPINE2,Ccl9,CSF2RB,ITGB2,F9,CYLD,CYR61,
Molecular Transport	1.6E-05-7.96E-03	ANK3,IGFBP4,S100A6,PTGER3,SLC36A2,CCL5,ATP2A2,TGM2,SLC30A7,CCL13,GAL,KLF2,PTPRN,DARC,SLC12A6,NPY,PDE10A,PDYN,CARTPT,HDC,MAPK8,PGAM2,UGT8,RAMP1,CNKSR3,PLLP,ITGAL,INHBA,SLC34A3,CLEC7A,MYOC,PCSK1,CCL7,CEBPD,P2RY12,ADCYAP1,AGTR2,ARG1
	1.1E-04-5.95E-03	CSF2RB,RAC2,ITGB2,Ly6a (includes others),RARRES2,F2R,TGFB1,C5,Ccl6,ESR1,Ccl9
Hematological System Development and Function	1.99E-05-7.91E-03	SOCS3,F2RL2,PKNOX1,SPN,PTGER3,LTB,ALOX12,CCL5,ATP2A2,SERPIND1,TGM2,CCL13,GAL,FOSL1,CCRL1,KLF2,PTPRN,DARC,ETS1,NPY,ATF3,CARTPT,HDC,MAPK8,IER3,RAMP1,GAB3,ITGAL,INHBA,CLEC7A,PCSK1,CCL7,CEBPD,BTG2,P2RY12,SBNO2,RHCE/RHD,ADCYAP1,JAK3,CTTN,CD34,AGTR2,ARG1
	3.77E-06-9.03E-03	RAC2,Ly6a,RARRES2,F2R,PYCARD,ZFP36L1,RAPGEF6,HRG,GRAP,CD52,IKZF2,TGFB1,LYL1,IFI30,CD274,Ccl6,BST1,ETS1,PTPRCAP,VWF,MAN2A1,C5,ARHGDIB,SERPINE2,Ccl9,CSF2RB,ITGB2,F9,CYLD,CYR61,ESR1
Immune Cell Trafficking	1.99E-05-7.27E-03	SOCS3,SPN,PTGER3,LTB,ALOX12,CCL5,TGM2,CCL13,FOSL1,KLF2,DARC,NPY,ETS1,ATF3,HDC,MAPK8,IER3,ITGAL,INHBA,CLEC7A,CCL7,SBNO2,JAK3,ADCYAP1,CD34,CTTN,ARG1
	6.34E-06-9.03E-03	ETS1,RAC2,RARRES2,Ly6a (includes others),F2R,PYCARD,RAPGEF6,VWF,C5,HRG,Ccl9,CSF2RB,ITGB2,TGFB1,CD274,ESR1,Ccl6
Inflammatory Response	2.16E-05-7.93E-03	,INHBA,CLEC7A,PCSK1,CCL7,SBNO2,ADCYAP1,JAK3,CD34,CTTN,AGTR2,ARG1
	1.87E-06-9.03E-03	RAC2,Ly6a,RARRES2,F2R,PYCARD,TP73,HRG,TGFB1,CYBA,LYL1,CD274,Ccl6,ETS1,VWF,C5,MAN2A1,OGG1,Ccl9,SERPINE2,ITGB2,CSF2RB,RETNLB,SREBF2,F9,CYLD,ESR1,CHIA
Tissue Development	2.69E-05-7.39E-03	AKAP12,SOCS3,CADM3,F2RL2,SPN,PTGER3,LTB,ALOX12,CCL5,ATP2A2,LMNB1,ADAMTS2,HOXD10,SERPIND1,TGM2,CCL13,MGP,FOSL1,PCDH12,FZD2,KLF2,DARC,SLC12A6,ETS1,NPY,PARVA,ENPP1,CARTPT,HDC,MAPK8,IER3,Meg3,ITGAL,INHBA,SLC34A3,CLEC7A,MYOC,CCL7,LOXL1,P2RY12,RIMS1,ADCYAP1,RHCE/RHD,JAK3,CD34,CTTN,AGTR2,RPSA,ARG1
	1.28E-04-8.26E-03	ETS1,RAC2,CADM3,Ly6a,F2R,RAPGEF6,VWF,MAN2A1,C5,SERPINE2,ITGB2,CSF2RB,TGFB1,CD274,MAN2A2,CYLD,CYR61,ESR1,WNT11,LUZP1
Cellular Function and Maintenance	2.96E-05-7.25E-03	ANK3,AKAP12,FZD10,SOCS3,IGFBP4,F2RL2,PKNOX1,SPN,LTB,CCL5,ATP2A2,DUSP2,TGM2,CCL13,GAL,CENPE,CCRL1,EEF2K,KLF2,DARC,NPY,ETS1,PARVA,ATF3,ENPP1,CARTPT,HDC,MAPK8,UGT8,PYGL,ITGAL,INHBA,SLC34A3,AKAP2/PALM2-AKAP2,CLEC7A,PCSK1,PSD,CCL7,BTG2,P2RY12,RHCE/RHD,ADCYAP1,JAK3,CTTN,AGTR2
	2.41E-05-9.01E-03	RAC2,Ly6a,F2R,PYCARD,HRG,TGFB1,LYL1,IFI30,CD274,MSTO1,BST1,ETS1,PTPRCAP,SEC23IP,VWF,MT3,C5,MAN2A1,KIF3B,ARHGDIB,CSF2RB,ITGB2,DSCAM,CYLD,CYR61,ESR1,PPP1R9A
Cell-mediated Immune Response	5.21E-05-7.25E-03	ETS1,SOCS3,PKNOX1,SPN,HDC,MAPK8,LTB,CCL5,ITGAL,INHBA,TGM2,PCSK1,CCL13,CCL7,JAK3,ADCYAP1,KLF2,DARC
	2.67E-04-7.06E-03	ETS1,ITGB2,CD52,Ly6a (includes others),TGFB1,CD274,CYLD
Cellular Development	5.21E-05-7.39E-03	CSTA,SRSF2,IGFBP4,SOCS3,PKNOX1,SPN,LTB,ALOX12,CCL5,LMNB1,LOXL2,ADAMTS2,SERPIND1,TGM2,DDX5,CCL13,GAL,MGP,CCRL1,FOSL1,FZD2,KLF2,PTPRN,SLC12A6,ETS1,NPY,ENPP1,ATF3,CARTPT,MAPK8,PGAM2,UGT8,GAB3,ITGAL,INHBA,ASPA,PCSK1,CEBPD,BTG2,P2RY12,JAK3,ADCYAP1,CD34,AGTR2,ARG1
	1.28E-04-8.44E-03	RAC2,Ly6a,RARRES2,F2R,ELAVL3,PYCARD,TP73,LRG1,ZFP36L1,NPNT,GRAP,IKZF2,TGFB1,LYL1,OSMR,CD274,ATN1,ETS1,PTPRCAP,PELP1,VWF,DKKL1,C5,ARHGDIB,Ccl9,SERPINE2,CSF2RB,ITGB2,CYLD,CYR61,ESR1,WNT11
Lipid Metabolism	5.21E-05-7.25E-03	NPY,SOCS3,IGFBP4,JAZF1,CARTPT,HDC,MAPK8,UGT8,ALOX12,CCL5,ITGAL,INHBA,ASPA,CLEC7A,CCL13,GAL,LPCAT1,CEBPD,ADCYAP1,AGTR2
	1.1E-04-5.95E-03	CSF2RB,RARRES2,F2R,TGFB1,C5,ESR1
Cell Morphology	5.88E-05-7.39E-03	K3,ADCYAP1,CTTN,AGTR2,ARG1
	2.83E-04-5.95E-03	ETS1,RAC2,Ly6a,F2R,TP73,SEC23IP,VWF,MT3,MAN2A1,C5,KIF3B,SERPINE2,ITGB2,TGFB1,CD274,OSMR,DSCAM,MAN2A2,CYLD,ESR1,WNT11,PPP1R9A
Hypersensitivity Response	5.88E-05-7.25E-03	ATF3,CCL13,CCL7,HDC,CCL5,JAK3
	2.04E-04-3.57E-03	RAC2,ITGB2,F2R,TGFB1,C5
