# Supplementary figures and images for: Acute and chronic phases of complex regional pain syndrome in mice are accompanied by distinct transcriptional changes in the spinal cord
Source: Mol Pain. 2013 Aug 8;9:40. doi: 10.1186/1744-8069-9-40 (PMC3751593; doi:10.1186/1744-8069-9-40)

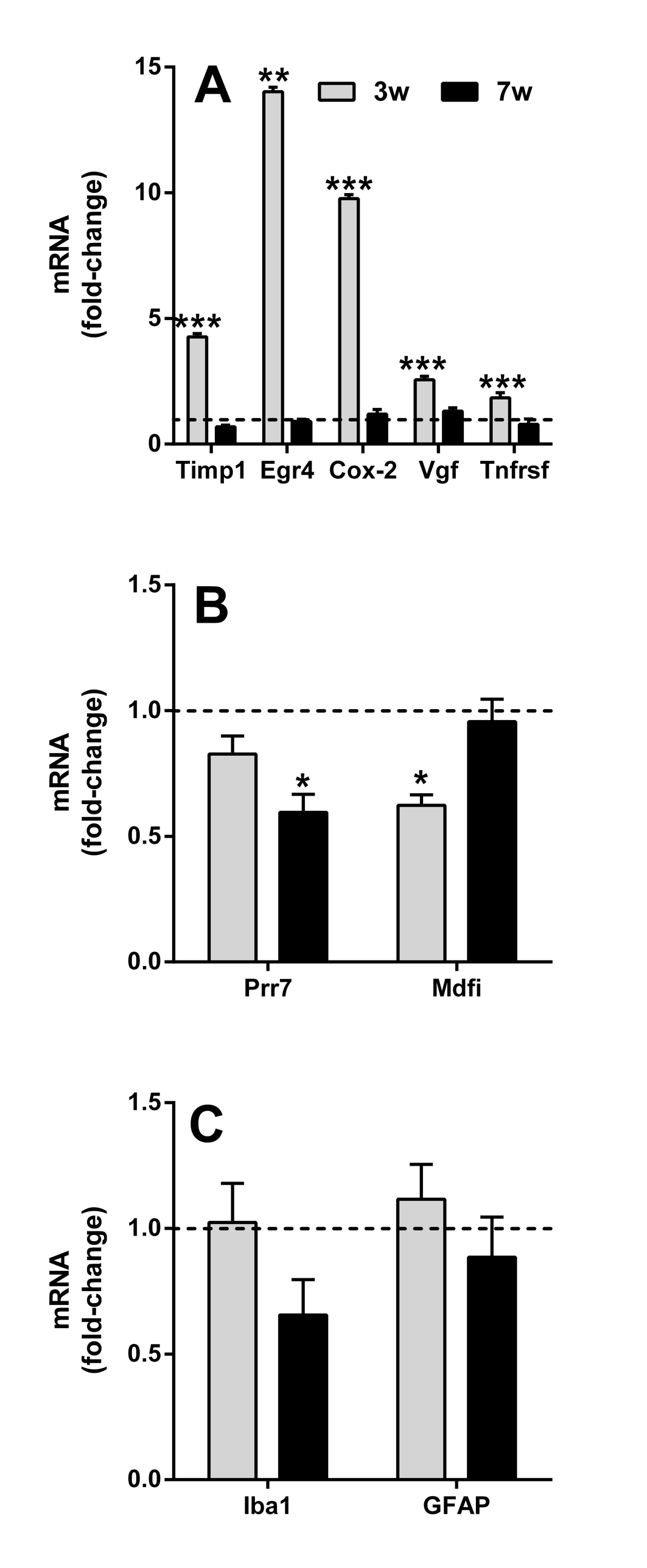

Supplement: Additional file 4: Figure S1 — Validation of transcript mRNA expression. qPCR validation of upregulated (A), downregulated (B), and unchanged (C) transcripts in the ipsilateral spinal cord 3 and 7 weeks after fracture. The dotted line indicates control measures. * p<0.05. n=4/group. Errors bars=S.E.M. [file 1744-8069-9-40-S4.jpeg]

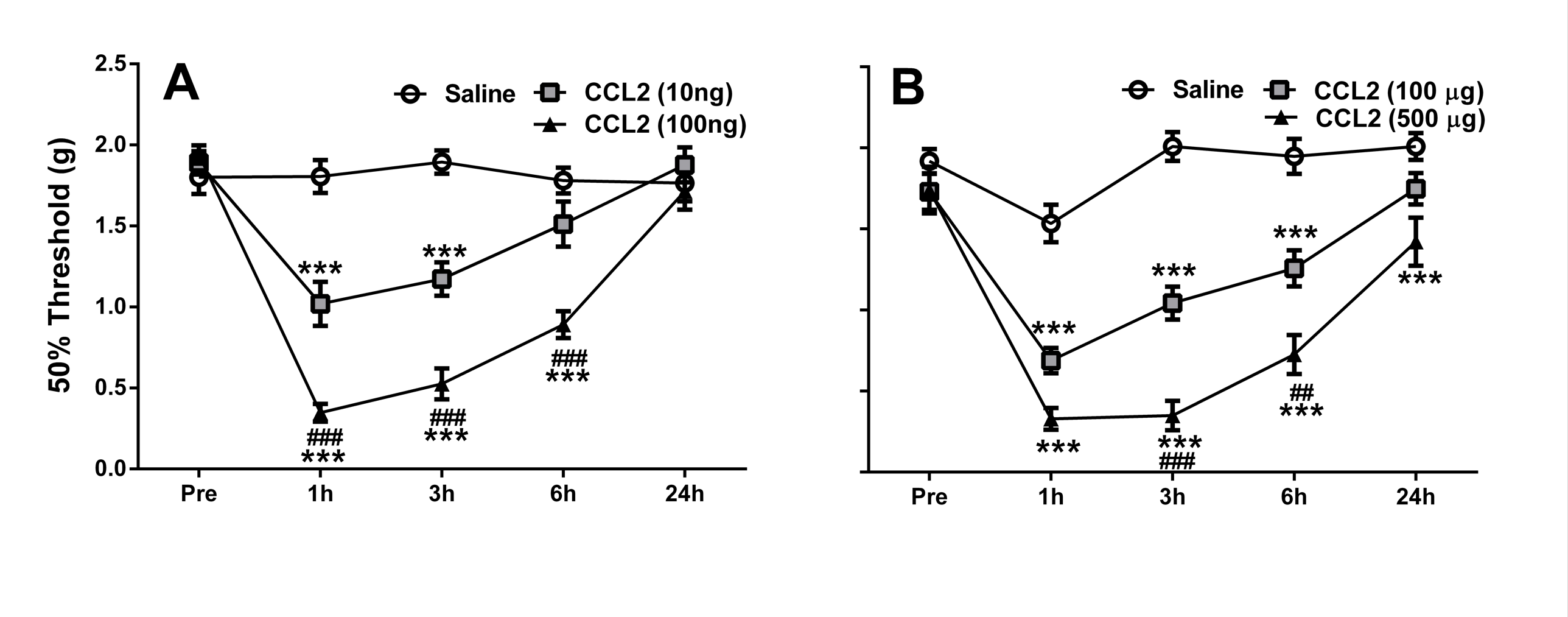

Supplement: Additional file 5: Figure S2 — Induction of mechanical allodynia following the intrathecal and intraplantar administration of CcL2. Both intrathecal (A) and intraplantar (B) administration of CcL2 resulted in decreased mechanical thresholds compared to vehicle-treated mice. *** p<0.001. n=7-8/group. Errors bars=S.E.M. [file 1744-8069-9-40-S5.jpeg]
